# Depletion of serotonin in the basolateral amygdala elevates glutamate receptors and facilitates fear-potentiated startle

**DOI:** 10.1038/tp.2013.66

**Published:** 2013-09-03

**Authors:** L Tran, B K Lasher, K A Young, N B Keele

**Affiliations:** 1 Institute for Biomedical Studies, Baylor University, Waco, TX, USA; 2 Department of Psychology and Neuroscience, Baylor University, Waco, TX, USA; 3 Department of Psychiatry and Behavioral Sciences, Texas A&M Health Science Center College of Medicine, Temple, TX, USA; 4 Neuropsychiatry Research Program, Central Texas Veterans Health Care System, Temple, TX, USA

**Keywords:** anxiety, 5,7-DHT, emotions, fear, GluR1, 5-HT

## Abstract

Our previous experiments demonstrated that systemic depletion of serotonin (5-hydroxytryptamine, 5-HT), similar to levels reported in patients with emotional disorders, enhanced glutamateric activity in the lateral nucleus of the amygdala (LA) and potentiated fear behaviors. However, the effects of isolated depletion of 5-HT in the LA, and the molecular mechanisms underlying enhanced glutamatergic activity are unknown. In the present study, we tested the hypothesis that depletion of 5-HT in the LA induces increased fear behavior, and concomitantly enhances glutamate receptor (GluR) expression. Bilateral infusions of 5,7-dihydroxytryptamine (4 μg per side) into the LA produced a regional reduction of serotonergic fibers, resulting in decreased 5-HT concentrations. The induction of low 5-HT in the LA elevated fear-potentiated startle, with a parallel increase in GluR1 mRNA and GluR1 protein expression. These findings suggest that low 5-HT concentrations in the LA may facilitate fear behavior through enhanced GluR-mediated mechanisms. Moreover, our data support a relationship between 5-HT and glutamate in psychopathologies.

## Introduction

Despite the growing prevalence of emotional disorders in modern society, the underlying pathophysiology is still unknown. However, clinical and preclinical evidence implicate neuronal hyperexcitability in the amygdala as a pivotal factor contributing to emotional disturbances, such as pathological fear, aggression and anxiety.^1, 2, 3^ When epileptic foci, characterized by hyperexcitable neurons, are located in the amygdala, patients often report comorbid emotional disturbances during the interictal period. Experiments in animal models also show that kindling-induced hyperecitability in the amygdala can potentiate abnormal emotional behaviors.^4^ This evidence supports the possibility that emotional disorders involve neuronal hyperexcitability in the amygdala.

Many phenotypes can facilitate neuronal hyperexcitability such as low serotonin (5-hydroxytryptamine; 5-HT), a hallmark characteristic of patients with emotional disorders.^5^ Previous studies in rodent models demonstrate that systemic depletion of 5-HT can induce functional changes in the amygdala, including increased excitatory post-synaptic potentials and burst firing.^6, 7^ Moreover, animals with lower 5-HT levels exhibit increased aggressive behaviors and enhanced fear-potentiated startle (FPS).^1, 8^ These studies suggest that low 5-HT facilitates abnormal emotional behavior through an induction of neuronal hyperexcitability in the amygdala.

Excitability in the amygdala is mediated by glutamatergic signaling, and is enhanced under various conditions.^9, 10, 11^ Glutamatergic signaling can be strengthened by increased glutamate receptor (GluR) expression, which has been observed in kindled animals.^12, 13, 14^ Importantly, when GluRs are overexpressed in the amygdala of animals, there is an elevation of abnormal behaviors,^15^ suggesting that an upregulation of GluRs may mediate low 5-HT-induced abnormal behaviors. In support, our preliminary experiments show that systemic depletion of 5-HT can enhance expression of GluR in the amygdala.^16^

An important determinant that remains to be investigated is whether depletion of 5-HT acts directly in the amygdala, or indirectly through connecting brain regions that input onto the amygdala such as the hippocampus or sensory cortex. Therefore, the aim of the present study was to investigate whether selective depletion of 5-HT in the amygdala would elevate fear responses, anxiety-like behavior and expression of GluRs in the amygdala.

## Materials and methods

### Animals

All experimental animal procedures were conducted in accordance with the Guide for the Care and Use of Laboratory Animals and conformed to a protocol approved by Baylor University Institutional Animal Care and Use Committee. Male Sprague–Dawley rats, approximately 150 g upon arrival (*n*=60; Harlan, Houston, TX, USA) were group-housed in a light-controlled, 12-h light/dark cycle and temperature-controlled (23 ^°^C) room. Commercial rodent pellets and water were provided *ad libitum*. All animals were allowed to acclimate to the facility at least 1 week before experimentation.

### Stereotaxic lesion of 5-HTergic fibers

5′7-Dihydroxytryptamine (5,7-DHT) selectively lesions local serotonergic fibers when combined with systemic treatment with desipramine.^17, 18^ Animals received pre-surgical intraperitoneal (i.p.) injections of desipramine (30 mg/kg) 30 min before anesthesia to protect norepinephrine transporters. Animals were then anesthetized with i.p. injections of equithesin (25 mg kg^−1^ pentobarbital, 150 mg kg^−1^ chloral hydrate). Using a stereotaxis device (Kopf Instruments, Tujunga, CA, USA) and aseptic technique, a midline incision was made and 1.5 mm holes were drilled in the skull −2.7 mm posterior to bregma and ±4.7 mm lateral from the midline. The tip of a 1-μl 22-G Hamilton microsyringe (Hamilton Co., Reno, NV, USA) was lowered 6.5 mm from the skull surface into the lateral amygdala (LA). Rats receive bilateral infusions of either 5,7-DHT (8 μg/ml) or vehicle (VEH) (1% ascorbic acid/saline) at a rate of 0.5 μl over 2.5 min as previously described.^19^ The needle remained in place for an additional 5 min following infusion. Animals were individually housed following surgery to prevent post-surgical complications, and allowed to recover for 1 week before experiment initiation.

### FPS

The FPS protocol was conducted as previously described.^20, 21^ On days 1 and 2, the rats were acclimated to the testing chambers (Acoustic Startle Reflex System, Med Associates Inc., St Albans, VT, USA) for 30 min followed by habituation. Habituation sessions consisted of 30 pseudo-random exposures to 90, 95 or 105 dB white noise bursts (30–50 ms duration) with an interstimulus interval of 30 s. On day 3, rats received fear-conditioning training. In the same startle chambers, the animals received 10 pairings (CS-US) of an 80-lux fluorescent light (3.2 s) with a rapid onset of 15 μs, immediately followed by a 0.6-mA foot shock (50 ms) with a variable 2–4 min interstimulus interval. FPS testing occurred on day 4. Rats initially received five exposures of each dB startle in pseudo-random order (NA1). During the next block, 50% of the startles were paired with the light cue (CS+). Finally, a second block of startle stimuli was delivered identical to NA1 (NA2). There was an interstimulus interval of 30 s between each NA1, CS+ and NA2 presentations. The startle response was measured by an accelerometer and quantified as the baseline-subtracted amplitude of the first peak (deflection) within 50 ms of the startle noise burst. Measurements were recorded by a computer and stored for later analysis (Startle-FPS Pro Series, Med Associates Inc.). FPS was calculated as the increase in startle amplitude during the presence of a light (CS+) normalized to the startle amplitude obtained from the average of the noise alone NA1 and NA2 trials (CS−) and expressed as a percentage. Only amplitudes in response to 95 dB white noise bursts were analyzed to limit ceiling effects.

### Elevated-plus maze (EPM)

Anxiety-like behavior was assessed on the EPM as previously described.^22^ The procedure room was approximately 23 °C and was dimly lit. Animals were allowed to acclimate to the room for 30 min and then placed in the center of the EPM facing an open arm. Each rat was allowed to explore for 5 min while being recorded by a ceiling-mounted camera, and the video footage was analyzed by an investigator blind to treatment. The percentage of time spent in the open arms and the number of open arm entries were used to quantify anxiety-like behavior, with decreased open arm exploration and entries indicating higher anxiety.

### Sample collection

Rats were euthanized by rapid decapitation with an animal guillotine. Brains were washed in cold phosphate-buffered saline (2–4 ^°^C) and 2 mm slices containing the amygdala at 2.5 mm posterior to Bregma were dissected. Bilateral tissue-punches of 1 mm diameter were isolated from the amygdala, dorsal hippocampus and overlying cortex using a 1.0-mm diameter core sampler. The samples were weighed, flash frozen with dry ice/EtOH and stored at −80 °C for subsequent analysis. Rats used for histology were anesthetized with equithesin (35 mg kg^−1^ sodium pentobarbital; 145 mg kg^−1^ chloral hydrate) followed by transcardial perfusion of phosphate-buffered saline and 4% paraformaldehyde in phosphate-buffered saline for 5 min each. Whole brains were extracted and post-fixed overnight in 4% paraformaldehyde at 4 °C. The brains were then incubated overnight in Dulbecco's phosphate-buffered saline+sodium azide followed by cyroprotection in 10% sucrose, 20% sucrose and finally 30% sucrose. The brains were sectioned at 20 μm on a cryostat sliding microtome and mounted on slides.

### Immunohistochemistry

The tissue slices were hydrated with tris-buffered saline (TBS). Endogenous peroxidases were blocked (30% H_2_O_2_/MetOH) and the tissue was permeablized (100 mM lysine, 0.1% Triton X and 10% goat serum). The slides were washed in TBS and incubated in mouse anti-serotonin transporter (SERT) (Chemicon, Temecula, CA, USA) in 10% goat serum overnight. The following day, the slides were incubated with goat anti-mouse conjugated with HRP (Millipore, Bellerica, MA, USA) followed by TBS washes. The slides were processed with 3,3′-diaminobenzidine/Ni/H_2_O_2_ and dehydrated through graded EtOH, cleared with xylene and coverslipped with permount.

### High-performance liquid chromatography (HPLC)

HPLC was performed as previously described.^23^ Tissue samples were first protonized with 0.4 M HClO_4_ and 2 μM 2,3-dihydroxybenzoic was added as an internal standard. The samples were homogenized and centrifuged at 14 000 *g* for 15 min at 4 °C. The supernatants were collected and analyzed by HPLC coupled with electrochemical detection using a ESA CoulArray system (Thermo Scientific, Sunnyvale, CA, USA) containing two pumps with a flow of 0.420 ml min^−1^. The mobile phase contained 0.05 mM potassium phosphate, 8.5 mg/50 ml octylsulfate and 14% methanol (pH 2.65). A C_18_ reverse-phase column was used to separate neurotransmitters. Standards prepared were 100 μM of 3,4-dihydroxyphenylacetic acid (DOPAC), 3,4-dihydroxyphenethylamine (dopamine), 5-hydroxyindoleacetic acid and 5-HT.

### Western blot analysis

Tissue samples were homogenized in 20 volumes of homogenizing buffer (50 mM Tris-HCl (pH 7.4), 1 mM EDTA), supplemented with a protease inhibitor cocktail containing 104 mM 4-(2-aminoethyl) benzenesulfonyl fluoride hydrochloride, 0.08 mM aprotinin, 2.1 mM leupeptin, 3.6 mM bestatin, 1.5 mM pepstatin A and 1.4 mM E-64 (Sigma, St Louis, MO, USA). Samples were centrifuged at 5000 *g* for 5 min. The membrane fractions were reconstituted with 20 volumes of lysis buffer (50 mM Tris-HCl, pH 7.4, 1% NP-40, 0.25% sodium deoxycholate, 150 mM NaCl, 1 mM EDTA, 1 mM phenylmethylsulfonyl fluoride, 1 mM Na_3_VO_4_ and 1 mM NaF) with protease cocktail and incubated on ice for 30 min followed by centrifugation at 14 000 *g* for 20 min. The supernatant was collected and the protein concentration was determined by Bradford assay (Millipore). Approximately 30 μg of protein was separated on a 4–20% gradient polyacrylamide gel (Bio-Rad, Hercules, CA, USA) and transferred to poly(vinylidene fluoride) membrane (Millipore) using a wet transfer system (Bio-Rad). The membranes were blocked with 3% nonfat milk in TBS for 1 h followed by incubation with rabbit anti-GluR1-4 (Chemicon) overnight at 4 °C. Membranes were washed with TBS and incubated with HRP-conjugated goat anti-rabbit antibody (Millipore) for 30 min. Following the washes, bands were developed using an ECL Western Blot Detection Kit (Amersham, Piscataway, NJ, USA).

### Quantitative reverse transcriptase-PCR (qRT-PCR)

Quantification of mRNA expression was performed as previously described.^16^ Whole-cell RNA was extracted using TRI Reagent (Molecular Research Center, Cincinnati, OH, USA) and concentration of total RNA was determined by spectrophotometry (*λ*=260 nm). Extraction was followed by cDNA synthesis and real-time PCR using Dynamo SYBR Green 2-step qRT-PCR kit (NEB, Ipswich, MA, USA) in a total reaction volume of 25 μl. Primers for GluR analysis were obtained from integrated DNA Technologies (Corraville, IA, USA). A common primer that recognizes all GluR subunits and a specific primer for GluR1 were used. The common GluR primer was fwd: 5'-TCGTACCACCATTTGTTTTTCA-3' and the GluR1 primer was rev: 5'-AAGAGGGACGAGACCAGACAAC-3'. Primers for 18S (Maxim Biotech, San Francisco, CA, USA), used for normalization, were sense: 5′-CCGCAGCTAGGAATAATGGAATAGGAC-3′ and antisense: 5′-GTTAGCATGCCGAGAGTCTCGTTC-3′ (Maxim Biotech). The reaction was performed on a Corbett Rotor-Gene 6000 (Qiagen, Valencia, CA, USA) with the initial denaturation at 95 °C for 15 min, followed by 40 cycles of denaturing at 94 °C for 10 s, annealing at 59.4 °C for 30 s and extension 72 °C for 30 s, and a final extension at 72 °C for 10 min. Changes in GluR1 transcripts were quantified using the comparative ΔΔ*C*_(T)_ method.

### Experimental design

Rats were assigned to one of two treatment groups, 5,7-DHT or VEH, and received stereotaxic infusions accordingly. Rats were further subdivided into three separate groups that were either subjected to fear-conditioning protocol, anxiety assay or euthanized for biochemical and molecular assays. Because of the influence of FPS on GluR1 expression,^24^ animals used for molecular assays were not exposed to behavioral assays. The brains of animals subjected to behavioral testing were used for SERT immunohistochemistry. An amygdala tissue sample was isolated from each brain hemisphere of the remaining animals that did not undergo behavioral testing. One sample was used for qRT-PCR, and the other sample was used for HPLC and western blotting. The assay assignment for each sample was randomized to control for possible lateralization effects.

### Statistical analysis

A Student's unpaired *t*-test was used to analyze biochemical and molecular experiments for significance. A one-way analysis of variance (ANOVA) was used to analyze behavioral data followed by a Tukey-Kramer's *post-hoc* test to compare the means. Grubbs' test was used to detect significant outliers, which were removed from further data analysis. A Pearson product-moment correlation coefficient was calculated followed by regression analysis, to determine strength of the relationship between 5-HT concentration and GluR1 mRNA and protein levels. The means and SEM are reported and significance was analyzed using GraphPad Prism Software (Ver 4.3, LaJolla, CA, USA), where significance was defined as *P*<0.05.

## Results

### 5,7-DHT treatment decreased 5-HTergic fiber density and reduced 5-HT levels

**Figure 1:**
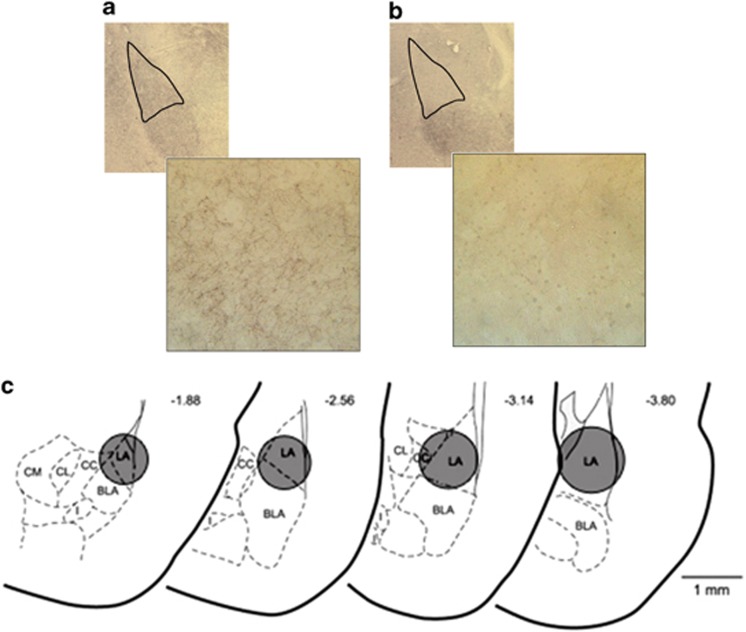
5,7-Dihydroxytryptamine (5,7-DHT) infusion decrease serotonergic fibers innervating the lateral amygdala (LA). (**a**) Visualization of serotonin transporter (SERT) immunoreactivity ( × 2.5 magnification) highlights serotonergic fibers innervating the amygdala. The LA is demarcated (top). At × 40 magnification, individual 5-HTergic fibers are clearly discernable (bottom). (**b**) SERT immunoreactivity was greatly reduced in 5,7-DHT-infused animals. Fibers are still intact in the basal nucleus of the amygdala, amygdalostriatal transition area and central nucleus of the amygdala (top). Within the diffusion radius, there is a clear reduction in the number of fibers (bottom). (**c**) A summary of the diffusion radius is shown. CC, central nucleus of the amygdala, capsular division; CL, central amygdala nucleus, lateral division; CM, central nucleus of the amygdala, medial division.

The serotonergic fibers were visualized by 5-HT transporter (SERT) immunostaining. As shown in the photomicrograph (Figure 1a), animals treated with VEH had robust positive SERT staining in the LA. Following 5,7-DHT treatment, there was a qualitative decrease in 5-HTergic fibers, which was constrained to the infusion area at coordinates posterior −2.7 mm and lateral 4.8 mm from bregma, and depth 6.7 mm measured from the skull (Figure 1b). The size of the 5-HTergic fiber reduction was approximately 1 mm in diameter and extended slightly beyond the LA to include adjacent areas of the central amygdala, basal amygdala and portions of the dorsal striatum.

### Microinjections of 5,7-DHT into the LA decreased 5-HT

Depletion of 5-HT in the LA was verified by HPLC analysis. Analysis of monoamines and metabolites showed that 5,7-DHT infusions selectively reduced 5-HT by an average of 43.4±6.7% (*P*=0.0004) and its primary metabolite 5-hydroxyindoleacetic acid by an average of 27.9±9.8% (*P*=0.03) in comparison to VEH-infused animals (Table 1). There was no significant treatment effect on dopamine (*P*=0.34) or its metabolite DOPAC in the amygdala (*P*=0.09). There were no significant changes in analyzed monoamines or metabolites in the hippocampus or cortex (*P*>0.10), confirming region specificity.

### 5,7-DHT treatment increases FPS

**Figure 2:**
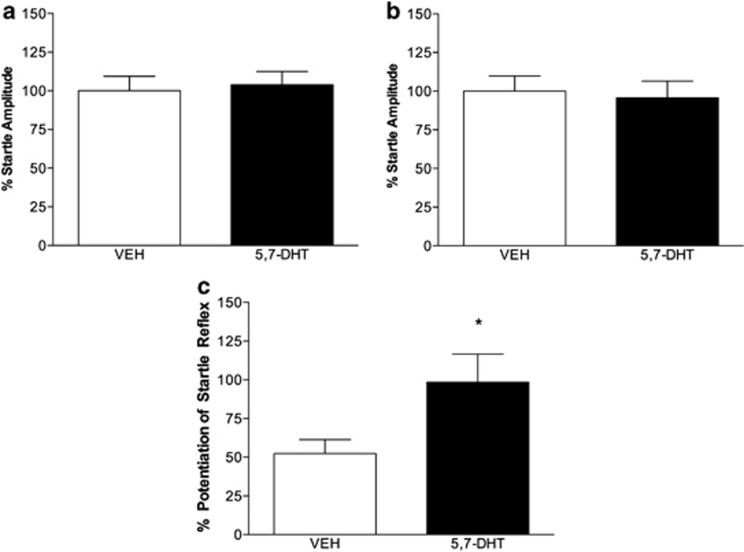
5,7-Dihydroxytryptamine (5,7-DHT) infusions into the amygdala increase fear-potentiated startle (FPS). (**a**) During habituation, acoustic startle reflex to noise alone was unchanged by amygdala 5,7-DHT treatments. (**b**) The startle response to 95 dB noise alone recorded during training was also unchanged by intra-amygdala infusion of 5,7-DHT. (**c**) In VEH-infused rats (*n*=15), the FPS reflex was lower than animals treated with 5,7-DHT infusions (*n*=18). Data represents mean±s.e.m., and *P<0.05 by one-way analysis of variance.

Treatment with 5,7-DHT did not have a significant effect on baseline startle, and the startle amplitudes for 5,7-DHT animals to 95 dB startle stimuli during habituation was 104±9% compared with VEH controls at 100±36% (Figure 2a); *P*>0.05. On test day, 5,7-DHT had startle amplitudes of 96±11% normalized in comparison to VEH-treated animals at 100±10% (Figure 2b); *P*>0.05. Following FPS training, both 5,7-DHT-infused and VEH-infused rats showed FPS (Figure 2c), but there was a significant effect of 5,7-DHT treatment on startle amplitudes (F_(1, 31)_=4.522, *P*<0.05). FPS amplitude was increase to 198±18% in rats treated with intra-amygdala infusions of 5,7-DHT in comparison to VEH-infused control rats (152±9%).

### 5,7-DHT treatment does not influence anxiety-like behavior

Because the amygdala is also involved in unconditioned fear, we evaluated the behavioral effects of low 5-HT on anxiety-like behavior on the EPM (Supplementary Figure 1). When exposed to the EPM, animals treated with 5,7-DHT averaged 4.5±1.5 open arm entries compared with VEH-treated animals (4.0±0.8; *P*=0.79). There was no difference in percent time spent in the open arms between 5,7-DHT-treated animals (20.8±9.5%) compared with VEH controls (16.7±5.2% *P*=0.64), indicating no difference in anxiety-like behavior.

### 5,7-DHT treatment increases GluR1 mRNA

**Figure 3:**
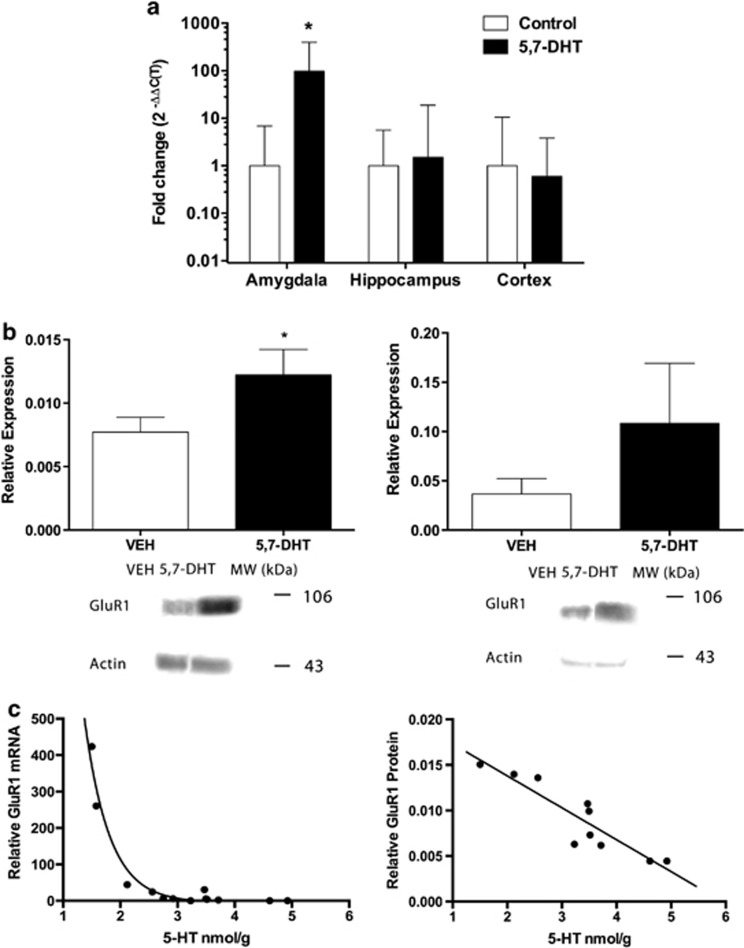
5,7-Dihydroxytryptamine (5,7-DHT) treatments increase glutamate receptor 1 (GluR1) expression in the amygdala. (**a**) There was a significant increase in GluR1 transcripts in 5,7-DHT-treated animals (*n*=10) compared with VEH-treated control animals (*n*=8) in the amygdala. No differences were seen in the hippocampus or cortex. Log transformed fold-changes in mRNA levels are expressed as mean±s.e.m., and *P<0.05 by unpaired Student's *t*-test. (**b**) Western blot analysis of tissue samples obtained from (left) the amygdala and (right) hippocampus of VEH-treated controls (*n*=5) and 5,7-DHT-treated animals (*n*=4) reveals positive GluR1 bands at ∼105 kDa. Representative blots are shown (bottom). GluR1 expression from each sample was normalized to β-actin bands at ∼43 kDa (GluR1/β-actin). Data represent mean±s.e.m., and *P<0.05 by unpaired Student's *t*-test. (**c**) A Pearson product-moment correlation coefficient was calculated to determine the relationship between 5-HT and GluR1 mRNA expression (*r*=−0.70; *P*=0.004) as well as protein expression (*r*=−0.91; *P*<0.001). Scatter plots are shown for (left) GluR1 mRNA and (right) GluR1 protein, and best-fit lines were calculated using nonlinear and linear regression, respectively.

In order to quantify the levels of GluR1 mRNA, the primary subunit comprising GluRs, samples collected from the amygdala were analyzed by qRT-PCR. Following 5,7-DHT treatment, there was an average 97.9-fold increase in GluR1 mRNA in the amygdala (Figure 3a). The normalized number of PCR cycles (relative to 18S rRNA) to detection threshold (Δ*C*_(T)_) for GluR1 transcripts in the amygdala was decreased from 3.98±2.40 cycles in samples from control to −2.64±2.02 in samples from 5,7-DHT-treated animals (*P*=0.03). There were no significant differences in Δ*C*_(T)_ values for hippocampus (VEH: 0.87±2.49, 5,7-DHT: 0.28±3.65; *P*=0.91) or cortex (VEH: 1.74±3.39, 5,7-DHT: 2.49±2.67; *P*=0.88).

### 5,7-DHT treatments increase GluR1 protein expression

To confirm changes in mRNA translate to protein expression, protein extracts from the amygdala were analyzed by western blot analysis. Results showed a 5,7-DHT treatment-associated increase in GluR1 protein expression in the amygdala (Figure 3b; left). In the hippocampus, 5,7-DHT treatments did not appear to affect the protein expression of GluR1 (right). Immunoreactive bands were further quantified and normalized to levels of β-actin. Results indicated an overall 58.8±0.26% increase in GluR1 expression in the amygdala of 5,7-DHT-treated animals compared with VEH controls (*P*=0.04). Although the mean of hippocampus GluR1 expression was higher in 5,7-DHT-infused animals, there was also an increase in variance, and the difference was not statistically significant (VEH: 0.04±0.02, 5,7-DHT: 0.11±0.06; *P*=0.24).

### Concentrations of 5-HT level negatively correlate with GluR1 expression

Concentrations of 5-HT were compared with both GluR1 mRNA expression and protein expression. The results indicated a negative correlation between 5-HT and GluR1 mRNA (*r*=−0.70; *P*=0.004) as well as protein levels (*r*=−0.91; *P*<0.001). Correlations are shown in Figure 3c, and curves were fitted using a nonlinear log-3 parametric function (*Y*=−4.967−4.967/1+10^(X+2.62)^; *R*^2^=0.96) for GluR1 mRNA and linear regression (*Y*=−0.004*X*+0.02; *R*^2^=0.83; F_(1,8)_=38.64; *P*=0.003) for protein expression.

### There was no effect of 5,7-DHT treatments on GluR2-4 protein expression

**Figure 4:**
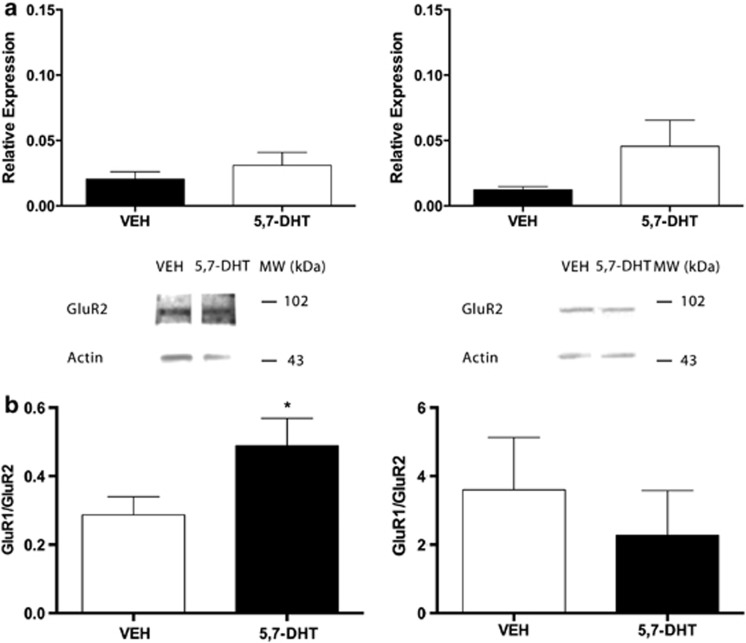
Glutamate receptor 2 (GluR2) protein expression is unchanged in the amygdala and the hippocampus following intra-lateral amygdala (LA) 5,7-dihydroxytryptamine (5,7-DHT) administrations. (**a**) Western blot analysis of tissue samples obtained from (left) the amygdala and (right) hippocampus of VEH-treated controls (*n*=5) and 5,7-DHT-treated animals (*n*=4) reveals positive GluR2 bands at ∼106 kDa. Representative blots show are shown (top). GluR2 expression from each sample was normalized to β-actin bands at ∼43 kDa (GluR2/β-actin). Data represent mean±s.e.m., and *P*>0.05 by unpaired Student's *t*-test. (**b**) Ratios between GluR1/GluR2 expression were calculated for the (left) amygdala and (right) hippocampus. Data represent mean±s.e.m. and *P<0.05 by unpaired Student's *t*-test.

Treatment with 5,7-DHT did not influence expression levels of GluR2 in either the amygdala or hippocampus in comparison to VEH controls (Figure 4a). Quantification of the optical densities confirmed levels of GluR2 in the amygdala were not significantly elevated (VEH: 0.02±0.01, 5,7-DHT: 0.03±0.01; *P*=0.36). Ratios of GluR1/GluR2 were calculated (Figure 4b), and results indicate an increase in ratio in the amygdala (*P*=0.04) following 5,7-DHT treatments (0.49±0.08) when compared with VEH-treated animals (0.29±0.05). In contrast, there was a trend for an increase in GluR2 subunits in the hippocampus (VEH: 0.01±0.002, 5,7-DHT: 0.05±0.02; *P*=0.09). Power analysis suggested that adding an additional two animals per group would improve the power to detect a significant difference with this effect size. When ratios between GluR1/GluR2 were calculated, there was no significant difference between treatment groups (VEH: 3.60±1.53; 5,7-DHT: 2.27±1.31; *P*=0.26). Analysis of GluR3 showed no significant differences between groups (VEH: 6.52±2.38; 5,7-DHT: 2.25±0.86; *P*=0.11), and analysis of GluR4 revealed similar results (VEH: 0.37±0.07; 5,7-DHT: 0.66±0.16; *P*=0.06).

## Discussion

The purpose of the present investigation was to examine the effects of isolated depletion of 5-HT in the LA on fear- and anxiety-like behavior, and to delineate the biological correlates involved. Using 5,7-DHT infusions into the LA, we selectively lowered 5-HT in the LA and observed enhanced FPS, but not anxiety-like behaviors. Depletion of 5-HT in the LA correlated with increased GluR1 mRNA and protein expression, indicating that low 5-HT may induce amygdala hyperexcitability by enhancing GluR expression. Overall, our results showed that reducing levels of 5-HT in the amygdala could potentiate amygdala glutamatergic neurotransmission and promote fear behaviors.

### Inducing low 5-HT in the LA

Previously, our lab demonstrated that systemic depletion of 5-HT facilitated fear learning, elevated amygdala excitatory post-synaptic potential and increased amygdala GluR1 transcription.^1, 6, 16, 21^ These and other findings support a pivotal role for perturbed amygdala 5-HT and enhanced glutamatergic mechanisms in the pathophysiology of emotional disorders. In the present study, we investigated the specific effect of depleting 5-HT in the LA on fear- and anxiety-like behaviors. Using 5,7-DHT microinjections into the LA, there was a robust reduction in amygdala serotonin, consistent with similar studies.^25, 26, 27, 28^ The 5-HT levels in the hippocampus and cortex were unaffected, and there was no change in dopamine or DOPAC in any brain region examined. Although our histological data showed that 5-HT depletion was most prominent in the LA, other areas were also affected including the CeA, which can influence startle and anxiety spectrum behaviors.^29^ Thus, although 5-HT depletion was effectively constrained to the LA, the interpretation of intra-amygdala localization of the observed effects is limited. However, as unconditioned startle amplitudes and anxiety observed on the EPM were not changed, the potential of 5,7-DHT influencing regions associated with unconditioned fear can be eliminated.

### The effect of decreasing 5-HT in the LA on fear and anxiety

Having established a reduction in 5-HT with 5,7-DHT treatments, our next goal was to examine the effects of low 5-HT in the LA on the cardinal behaviors regulated by the amygdala including fear and anxiety. Activation of 5-HT receptors in the LA inhibits the output because of the specific localization of the different subtypes.^7, 30^ A reduction in 5-HT decreases 5-HT_2A_ and 5-HT_3_-mediated excitation of inhibitory interneurons, and concomitantly attenuate 5-HT_1A_ and 5-HT_1B_-mediated inhibition of excitatory projection neurons. In result, we found that treatment with 5,7-DHT induced exaggerated fear responses measured in the FPS paradigm, which is consistent with our previous study that reported low systemic 5-HT could enhance fear learning.^21^ An important caveat to note is that our experiments do not fully distinguish whether the effects of low 5-HT influence mechanisms associated with acquisition or expression of fear. As the rats are infused before fear conditioning, the treatment could potentially influence mechanisms associated with acquisition and/or expression. However, previous studies investigating the role of GluR1 in fear conditioning have demonstrated that GluR1 regulation occurs predominantly at acquisition of fear memory.^31^ Together with our results, this suggests that low 5-HT-induced increase in fear learning is a result of increased GluR1 during the acquisition of fear memory. However, we cannot preclude the possibility of 5,7-DHT-induced changes in the expression of fear. In addition to conditioned fear behaviors, clinical data have implicated 5-HT involvement in anxiety spectrum behaviors.^32^ Furthermore, previous studies in animal models have reported a facilitation of anxiety-like behavior following systemic depletion of 5-HT.^33^ However, in contrast to the FPS effects resulting from a decrease in amygdala 5-HT, we observed no influences on anxiety-like behavior, which supports previous studies using similar 5,7-DHT infusions into the LA.^18^ Thus, the evidence from present study suggests that a decrease in 5-HT levels in the LA selectively facilitates abnormally heightened conditioned fear learning, but not unconditioned anxiety-like behaviors.

### The potential role of GluRs in facilitating low 5-HT-induced behaviors

The next aim was to examine potential biological correlates that may be responsible for the low 5-HT-enhanced fear behaviors observed. Specifically, we investigated GluR expression in the LA by quantifying changes of individual subunits in response to 5,7-DHT treatments. We observed an upregulation of GluR1 mRNA and protein expression in 5,7-DHT-infused animals, similar to our previous findings in the LA of animals with systemic depletion of 5-HT.^16^ The GluR1 expression levels correlated with the concentration of 5-HT in the LA, suggesting a significant relationship between 5-HT levels and GluR expression. In contrast to GluR1 subunits, we discovered that the expression level of GluR2 subunits, which decreases calcium permeability and attenuates excitability, were unchanged in the LA. The increase in GluR1 in the absence of an increase in GluR2 indicates an overall enhancement of neuronal excitability in the LA because of low 5-HT. We have suggested previously that including low 5-HT may enhance the degree of rectification of glutamatergic synaptic currents in the LA, and reflect a decrease in the relative contribution of GluR2-containing GluRs.^1^ Thus, our data suggest that low levels of 5-HT in the amygdala may facilitate neuronal hyperexcitability at least in part by upregulating GluR expression. Although the specific 5-HT receptor subtype(s) in the LA-mediating changes in GluR1 expression and exaggerated FPS are of interest, we did not attempt to delineate the role of 5-HT receptors in the present study. However, our previous investigation suggests that increased GluR1 and enhanced FPS following a decrease in 5-HT may be mediated by deficient 5-HT_2A_ signaling,^20^ and future experiments beyond the scope of the present study will test this hypothesis.

### Clinical implications

The present findings have important implications for the role of glutamate in regulating emotions. Studies in patients have suggested that GluR antagonist, such as ketamine, can be effective in treating mood disorders.^34^ Animal studies have also found the effects of ketamine to be comparable to traditional mood stabilizers,^35^ and similar results were observed with other GluR antagonists, including NPC 17742 and phencyclidine.^36^ In addition, 5,5-diphenylhydantoin (phenytoin), which can block GluR responses,^37, 38^ has been shown to be effective at treating anxiety,^37^ and dose-dependently reduces FPS in animals.^21^ Although the precise mechanism of these drugs in regulating emotions is not fully understood, in light of the data from the present study, it is possible that they block hyperexcitabilty at synapses that have been sensitized because of upregulation of GluR receptors. Moreover, the amygdala may be an area of the brain where these drugs can be expected to attenuate the effects of elevated GluR activity and restore normal circuit activity within the limbic system.

### Conclusions

Overall, our data increase the understanding of the molecular mechanisms of neuronal excitability in the amygdala, and identifies cellular changes that may contribute to fear-related behavioral alterations associated with reduced serotonergic signaling. We also provide evidence bridging the relationship between 5-HT and glutamate in regulating emotional behaviors. Furthermore, the identification of amygdala GluRs as a pivotal component contributing to amygdala hyperactivity after 5-HT depletion provides novel insight into the therapeutic efficacy of glutamateric agents that have shown potential in treating emotional disorders.

## Figures and Tables

**Table 1:** 5,7-DHT treatment decreases 5-HT levels in the LA

	*Amygdala*		*Hippocampus*		*Cortex*	
	*VEH*	*5,7-DHT*	*VEH*	*5,7-DHT*	*VEH*	*5,7-DHT*
5-HT	3.96±0.26	2.24±0.25***	1.39±0.12	1.25±0.21	1.23±0.24	0.99±0.09
5-HIAA	4.77±0.44	3.44±0.43*	2.49±0.16	2.32±0.24	1.85±0.34	1.41±0.16
DA	6.50±1.76	4.41±1.29	0.85±0.24	0.74±0.26	0.45±0.03	0.78±0.18
DOPAC	3.50±1.18	1.82±0.37	0.24±0.03	0.27±0.08	0.30±0.06	0.17±0.02

Abbreviations: 5,7-DHT, 5,7-dihydroxytryptamine; 5-HIAA, 5-hydroxyindoleacetic acid; 5-HT, 5-hydroxytryptamine; DA, dopamine; DOPAC, 3,4-dihydroxyphenylacetic acid. Amino levels are expressed as the mean concentration (nmolg^−1^ of tissue)±s.e.m. *P<0.05; ***P<0.001 by Student's unpaired *t*-test.
