# Supplementary figures and images for: Depletion of serotonin in the basolateral amygdala elevates glutamate receptors and facilitates fear-potentiated startle
Source: Transl Psychiatry. 2013 Sep 3;3(9):e298–. doi: 10.1038/tp.2013.66 (PMC3784761; doi:10.1038/tp.2013.66)

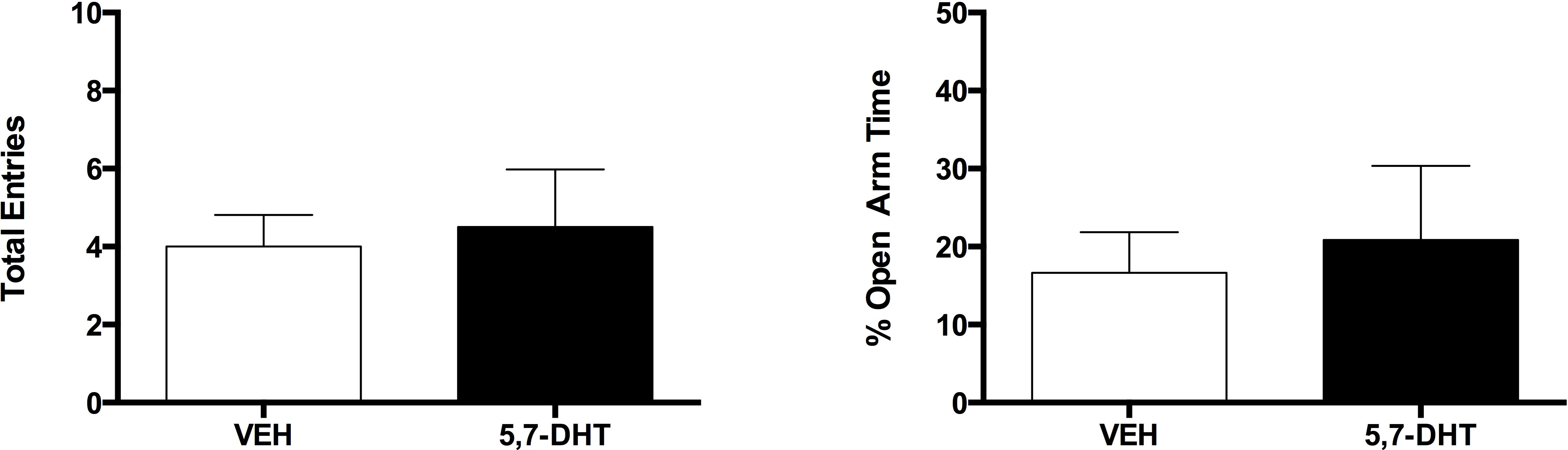

Supplement: Supplementary Figure 1 [file tp201366x1.tif]
