# Supplementary material for: Depletion of serotonin in the basolateral amygdala elevates glutamate receptors and facilitates fear-potentiated startle
Source: Transl Psychiatry. 2013 Sep 3;3(9):e298–. doi: 10.1038/tp.2013.66 (PMC3784761; doi:10.1038/tp.2013.66)
Supplement: Supplementary Figure Legend [file tp201366x2.doc]

**Supplementary Figure Legends**

*Supplemental Figure 1.* Anxiety-like behavior is not influenced by intra-LA administration of 5,7-DHT. Anxiety-like behavior was assessed on the EPM. Compared to VEH-infused rats (n=6), rats infused with 5,7-into the LA (n=6) resulted in no change in (A) open arm entries or (B) duration in the open arms. Data represents mean ± SEM, and *p* > 0.10 by unpaired Student’s t-test.
